# Body image, emotional intelligence and quality of life in peritoneal dialysis patients

**DOI:** 10.3934/publichealth.2023048

**Published:** 2023-08-14

**Authors:** Eleni Marki, Ioannis Moisoglou, Stamata Aggelidou, Maria Malliarou, Konstantinos Tsaras, Ioanna V. Papathanasiou

**Affiliations:** 1 Peritoneal Dialysis Unit, “Laiko” General Hospital of Athens, Athens, Greece and Hellenic Open University, Greece; 2 Department of Nursing, University of Thessaly, Larissa, Greece; 3 Nephrology Clinic, “Laiko” General Hospital of Athens, Athens, Greece; 4 Department of Nursing, University of Thessaly, Larissa, Greece and Hellenic Open University, Greece

**Keywords:** body image, emotional intelligence, patient, peritoneal dialysis, quality of life

## Abstract

**Background:**

End-stage-renal-disease is one of the most common chronic diseases, and peritoneal dialysis constitutes one of the replacement therapies. The aim of this study was to investigate the views of patients on peritoneal dialysis regarding their body image, to assess their quality of life and level of emotional intelligence.

**Methods:**

A cross-sectional study was performed with structured questionnaires. The sample of the study was the patients undergoing peritoneal dialysis and monitored by the nephrology clinics of 7 public hospitals in Greece.

**Results:**

A total of 102 completed questionnaires were collected and analyzed (68% response rate). The participants showed moderate degree of body-image dysphoria (mean = 1.29, *SD* = 0.94), moderate levels of emotional intelligence and experienced moderate quality of life. According to the statistical analysis, women reported worse body image (*p* = 0.013) and university graduates showed higher levels of emotionality (*p* = 0.016). The correlations between the quality of life questionnaire subscales and demographic characteristics revealed statistically significant relationships between marital status and the Physical Functionality subscale, where unmarried people had a better quality of life in this subscale (*p* = 0.042) and between postgraduate/doctoral degree holders and the subscale Patient Satisfaction (*p* = 0.035). Also, statistically significant relationships were found between occupation and the Social Interaction subscale, where those engaged in household activities and were unemployed (*p* = 0.022) showed better quality of life. Participants living in semi-urban areas had better quality of life on the subscale Burden of Kidney Disease (*p* = 0.034).

**Conclusion:**

ESRD patients on peritoneal dialysis suffer significant limitations related to disease and treatment modality. According to our findings, these affect both their body image as well as their quality of life. Improvement in emotional intelligence is the factor which plays an important mediating role in improving both body image and quality of life in patients on peritoneal dialysis.

## 1. Introduction

Chronic diseases are an important public health issue, affecting both individuals and health systems worldwide. Chronic diseases with a high prevalence include chronic kidney disease (CKD), which is a condition characterized by a gradual loss of kidney function over time. End-stage renal disease (ESRD), is the final, permanent stage of CKD, where the functionality of the kidneys has decreased to such an extent that the patient's life is endangered. The global estimated prevalence of CKD is 13.4% and patients with ESRD needing renal replacement therapy is estimated between 4.902 and 7.083 million [1]. In order to survive, patients suffering from ESRD need to go under dialysis or have kidney transplantation. According to The United States Renal Data System (USRDS), in year 2020, the predominant replacement therapy for new ESRD patients was hospital hemodialysis (83.9%), followed by peritoneal dialysis (12.7%), which is performed in the patient's home. Only a small percentage (3.1%) underwent kidney transplantation [2].

Due to the disease and its treatment, ESRD patients being on peritoneal dialysis experience a number of psychosomatic changes. One of these changes involves body image, which refers to an individual's perception of size, shape and aesthetics. The body image a person has is determined by personal history, attitudes and feelings regarding body weight and shape, cultural norms, psychological and biological factors. Body image may even affect a person's ability to carry out various activities [3]. It is common for patients on dialysis to be affected by edema development and by the presence of devices such as fistulas and catheters. These patients felt deformed, bloated and had a sense of unrecognizing themselves in the mirror [4]. Body-image disturbance in patients on dialysis (hemodialysis or peritoneal dialysis) is higher than in the general population. This is also associated with psychological morbidity development, in particular with anxiety and depression [5]. Increased levels of depression and anxiety in turn feed back into body image disturbance and reduce sexual satisfaction [6].

A chronic disease also affects the quality of a patients' life. Health-related quality of life is a complex concept and its assessment mainly concerns three dimensions of the patient's life [7]. First, it concerns the assessment of physical health, which refers to the person's ability to perform daily tasks and the physical symptoms resulting from the chronic disease. Second, it has to do with psychological functioning together with the well-being level of the individual and finally the social functioning, which relates to the patient's social integration and social relationships. In studies on the quality of life of ESRD patients on dialysis, comparisons have been made between the two treatment modalities, hemodialysis and peritoneal dialysis. Although, patients on peritoneal dialysis seem to experience better quality of life than those on hemodialysis, they also show a lower quality of life in the areas of burden and symptoms of kidney disease, worsened general health, emotional wellbeing and mental energy [8],[9].

An important skill for managing a chronic disease and its mental burden on the patient is emotional intelligence, which is defined as the “ability to monitor one's own and others' feelings and emotions, to discriminate among them and to use this information to guide one's thinking and actions” [10]. As ESRD patients on dialysis experience intense emotional stress characterized by depression and anxiety, they can achieve positive attitudes toward life through emotional intelligence skills, particularly “Motivating Emotions” [10]. The literature on the effect of emotional intelligence on CKD and ESRD is limited exclusively to patients undergoing hemodialysis. Studies regarding the role of emotional intelligence on the mental health of patients with CKD or in hemodialysis have shown that emotional intelligence mediates the relationship between uncertainty, anxiety and depression and helps patients to better manage posttraumatic growth [11],[12].

The aim of this study was to investigate the views of patients on peritoneal dialysis regarding their body image, to assess their quality of life and level of emotional intelligence.

## 2. Materials and methods

### 2.1. Study design and participants

A cross-sectional study was performed using structured questionnaires. The sample of the study was patients undergoing peritoneal dialysis and monitored by the nephrology clinics of 7 public hospitals in Greece. The study protocol was approved by the Ethical Committees of the hospitals under study. The study was conducted from 21st January 2021 to 31st May 2021. A total of 150 questionnaires were distributed and the method of collecting the questionnaires was the convenience sampling. Each patient was given an envelope, which included an informed consent with the researchers' contact details, the aim of the study and its ethical aspects (anonymity and voluntary participation).

### 2.2. Instruments

The Situational Inventory of Body-Image Dysphoria-Short Form (SIBID-S) scale was used to record the participants' views on body image [13]. For the present study, the Greek translated version was used [14]. SIBID-S is a 20-item scale scored on a 5-point Likert-scale (ranging from “Never” = 0 to “Always” or “Almost Always” = 4) and measures a person's negative body-image emotions (dysphoria) in specific situational contexts. Participants indicate how frequently they experience negative body image emotions in a variety of situations and contexts. The SIBID-S can be used in identifying situations that are especially distressing for persons with body-image difficulties and disorders. Higher scores indicating more frequent negative body image emotions.

The Greek version of The Trait Emotional Intelligence Questionnaire - Short Form (TEIQue-SF) was used to assess emotional intelligence [15]. The TEIQue-SF [16] is based on the full form of the TEIQue [17]. The scale consists of four subscales, Well-Being, Self-Control, Emotionality and Sociability. It includes 30 statements and participants are asked to respond to their level of agreement on a 7-point Likert-type scale (from “Strongly Disagree” = 1 to “Strongly Agree” = 7).

Kidney Disease Quality of Life - Short Form (KDQOL-SF) questionnaire was used to assess the health-related quality of life (HRQOL), which has been translated into Greek [18]. The KDQOL-SF is a validated instrument for quality of life assessment, which combines a disease-specific instrument (KDQOL) with a generic instrument (SF-36). The questionnaire consists of 23 questions covering 19 subscales. The items of the KDQOL-SF are scored on a Likert scale. Measured scores are linearly transformed into a scale ranging from 0 to 100. Higher scores indicate better HRQOL.

Also, we collected demographic data of the participants. These data include gender, age, marital status, educational level, occupational status, place of residence, partnership status and time on peritoneal dialysis.

### 2.3. Statistical analysis

The parametric t-test and One-Way ANOVA tests were used on the groups that met the basic conditions (Normality and equality of variances) for performing the parametric tests to check whether there were statistically significant differences between the groups of independent variables. A test was performed to determine whether there was a statistically significant relationship between the demographic characteristics of the patients and the scores of the scales studied. First, it was tested whether the observations in each control group followed a normal distribution using the Kolmogorov-Smirnov test, where the null hypothesis is formulated as “The distribution under test, does not differ from the normal distribution”. The equality of the variances between the groups of each independent variable was then tested using Levene's test, where the Null Hypothesis is formulated as “The variances of the two groups are equal”. The tests were performed at 0.05 level of significance, and therefore when the p-value (Sig.) was greater than 0.05, the null hypothesis could not be rejected. Where the p-value (Sig.) was less than the value of 0.05 in either the Kolmogorov-Smirnov or Levene's test, the null hypothesis was rejected, i.e., the conditions were not met by the groups and the non-parametric Mann-Whitney and Kruskall-Wallis tests were used to determine whether there was a statistically significant relationship between the groups of independent variables and the dependent variables. A 95% confidence level was set in all tests, i.e., a significance level of 0.05 was set. Also, a correlation analysis was performed between the scales of the 3 questionnaires used using Pearson's linear correlation coefficient. Data analysis was done with IBM SPSS 28.0 (Statistical Package for Social Sciences).

## 3. Results

### 3.1. Demographic characteristics

A total of 102 completed questionnaires were collected and analyzed (68% response rate). The demographic characteristics of the participants are shown in Table 1. The majority of participants belonged to the age group 40 to 60 years old (52.9%), were married (67.6%), secondary school / high school graduates (51%) and lived in an urban area (52.9%).

**Table 1.** Demographic characteristics of the participants (n = 102).

**Characteristics**	**Categories**	**n**	**%**
Gender	Male	50	49.0
	Female	52	51.0
Age	Up to 20 years old	1	1.0
	20 to 40 years old	9	8.8
	40 to 60 years old	54	52.9
	60 to 80 years old	37	36.3
	Over 80 years old	1	1.0
Time on peritoneal dialysis	Up to 1 year	27	26.5
	1 to 3 years	33	32.4
	3 to 6 years	31	30.4
	6 to 9 years	6	5.9
	9 to 12 years	4	3.9
	Over to 12 years	1	1.0
Marital status	Single	16	15.7
	Married	69	67.6
	Divorced	6	5.9
	Cohabitation	2	2.0
	Widowed	9	8.8
Educational level	Primary school	11	10.8
	Secondary school / high school	52	51.0
	University	34	33.3
	Postgraduate / doctorate	5	4.9
Occupational status	Civil servant	25	24.5
	Private employee	18	17.6
	Freelance	26	25.5
	Student	1	1.0
	Household	20	19.6
	Unemployed	12	11.8
Place of residence	Urban	54	52.9
	Semi-urban	31	30.4
	Rural	17	16.7
Partnership status	Alone	17	16.7
	With spouse	34	33.3
	With children	11	10.8
	Spouse and children	32	31.4
	Parents	8	7.8

### 3.2. Body image

The indicator for body image is presented in Table 2. The value of the Cronbach's alpha reliability index was calculated to be 0.948, indicating that there is high internal consistency reliability of the questions for the body image indicator. The maximum value of the body image index was 3.75 and the minimum was 0.00 while the mean value was about 1.29. According to this finding, the participants showed moderate degree of body-image dysphoria.

A correlation between the body image index and the demographic characteristics of the participants was then performed. According to the statistical analysis, only gender emerged as a statistically significant variable, with women reporting worse body image (*p* = 0.013).

### 3.3. Emotional intelligence

The descriptive results of TEIQue-SF are presented in Table 3. Participants showed moderate levels of emotional intelligence, with the Well-being subscale scoring the highest.

A correlation between TEIQue-SF and the demographic characteristics of the participants was then performed. A statistically significant relationship was found only for educational level, with university graduates showing higher levels of emotionality (*p* = 0.016).

**Table 2.** Descriptive analysis of the Body Image indicator (n = 102).

**Project**	**Value**
**Scale**	Body Image
**Min–Max**	0.00–3.75
**Mean ± *SD***	1.29 ± 0.94
**Cronbach's Alpha**	0.948

**Table 3.** Descriptive results of TEIQue-SF (n = 102).

**Scale**	**Min–Max**	**Mean ± SD**	**Cronbach's Alpha**
Well-Being	2.00–7.00	5.04 ± 1.22	0.770
Self-Control	2.00–7.00	4.58 ± 1.12	0.703
Emotionality	2.75–6.88	4.63 ± 0.90	0.672
Sociability	1.33–6.83	4.42 ± 1.19	0.725
Overall TEIQue-SF	2.53–6.73	4.68 ± 0.92	0.892

### 3.4. Quality of life

The descriptive results of KDQOL-SF are shown on Table 4. According to the findings, participants seem to experience a moderate quality of life.

The correlations between questionnaire subscales and demographic characteristics revealed statistically significant relationships with better quality of life: between marital status and the Physical Functionality subscale, where unmarried people had a better quality of life in this subscale (*p* = 0.042), between educational level, especially primary school graduates and the subscales Work (*p* = 0.016) and Cognitive Functioning (*p* = 0.005) and between postgraduate/doctoral degree holders and the subscale Patient Satisfaction (*p* = 0.035). Also, statistically significant relationships were found between occupation and the Social Interaction subscale, where those engaged in household and unemployed (*p* = 0.022) showed better quality of life. Participants living in semi-urban areas had better quality of life on the subscale Burden of Kidney Disease (*p* = 0.034). Meanwhile, on the General Health subscale, participants residing in semi-urban areas scored higher (*p* = 0.028).

**Table 4.** Descriptive results of KDQOL-SF (n = 102).

**Scale**	**Min–Max**	**Mean ± *SD***	**Cronbach's Alpha**
Symptom/problem list	31.2–95.3	71.57 ± 13.24	0.918
Effects of kidney disease	12.5–100	57.94 ± 17.17	0.963
Burden of kidney disease	0–100	50.49 ± 25.65	0.940
Work status	0–100	57.68 ± 36.69	0.932
Cognitive function	0–100	24.05 ± 23.36	0.911
Quality of social interaction	0–80	26.14 ± 18.80	0.940
Sexual function	0–100	43.14 ± 38.36	0.953
Sleep	52.5–75.0	64.88 ± 5.07	0.917
Social support	16.7–100	84.97 ± 19.41	0.975
Dialysis staff encouragement	6.2–100	79.29 ± 15.11	0.979
Patient satisfaction	0–100	80.07 ± 19.49	0.971
Physical functioning	5–100	63.38 ± 24.89	0.931
Role-physical	0–100	53.43 ± 43.24	0.938
Pain	0–100	73.51 ± 25.92	0.976
General health	10–100	49.80 ± 21.41	0.985
Emotional well-being	16–100	68.27 ± 19.05	0.981
Role-emotional	0–100	53.27 ± 39.60	0.892
Social function	0–100	61.03 ± 27.21	0.979
Energy/fatigue	20–75	55.98 ± 11.96	0.928

### 3.5. Association between body image, emotional intelligence and quality of life

Finally, the correlation between the three questionnaires was tested. Statistically significant relationships were found between quality of life and emotional intelligence. Specifically, statistically significant positive mean linear correlations were found between Mental Health and the subscales Well-being, Self-Control and total Emotional Intelligence with r = 0.510, r = 0.541 and r = 0.568 respectively. Also, the Vitality subscale showed statistically significant positive mean linear correlations with Well-being and overall Emotional Intelligence with correlation coefficients of 0.558 and 0.564 respectively.

## 4. Discussion

The present study highlighted that the patients on peritoneal dialysis experience moderate degrees of body-image dysphoria, moderate emotional intelligence and moderate quality of life. A chronic disease, through its symptoms, treatment and complications, can affect the patient's body image. Specifically, patients on peritoneal dialysis experience several challenges that can affect their body image and quality of life. These challenges include peritoneal catheter placement, risk of peritonitis and death, as well as modification of daily activities to comply with the peritoneal dialysis schedule [19],[20].

Positive body image has multiple protective benefits for the individual, as it is associated with well-being, self-care and adaptive physical health, whereas body image disturbance has the opposite effects [21]. Patients on both peritoneal and hemodialysis experience disturbance of their body image, with a significant effect on their psychological morbidity [22]. In this study, the burden of poor body image was found to be more strongly associated with women. This finding is consistent with the findings of studies showing women with ESRD or dialysis reporting worse body images [23] and greater body image anxiety [24]. Therefore, healthcare professionals should be aware of this important issue and provide psychological support to patients either alone or in collaboration with other healthcare professionals, such as a psychologist.

The quality of life of patients on peritoneal dialysis is poor, although it is better compared to that of patients on dialysis. The domains in which peritoneal dialysis patients have poorer quality of life are burden of kidney disease, physical health, general health, emotional wellbeing and energy/fatigue [25]. According to our study findings, patients with higher educational levels and those living in semi-urban areas have a better quality of life. These findings are consistent with those of other studies [25],[26]. Patients with higher levels of education are likely to have better knowledge regarding their ability to manage their treatment and its side effects, or because their educational level is related to their work, will experience a better quality of life (such as more social contacts and mobility, better mental health, etc.). Also, because of their disease management knowledge, it becomes more likely for them to have better collaboration with health professionals, while through collaboration they improve the quality of care and therefore report greater satisfaction with care, according to the findings of this study. Patients with a high level of education have better social functioning and lower chances of depression, and therefore experience a better quality of life [27]. Patients in semi-urban areas opt for peritoneal dialysis as hemodialysis requires travel to a hospital or hemodialysis unit usually located in a city. Living in semi-urban areas probably enables patients to have better social relationships or receive better social support, compared to those living in cities, and therefore have a better quality of life.

According to the statistically significant findings of the present study, those who are engaged in household work and are unemployed, as well as those who are unmarried, had a better quality of life. These findings are in contrast to those of studies of hemodialysis patients, which show that unemployed and unmarried people have worse quality of life [28]. This finding can be explained by the fact that hemodialysis and peritoneal dialysis have different characteristics. In particular, patients on peritoneal dialysis are treated three to five times a day. When patients are working, they are likely to experience difficulties in combining their work and treatment commitments, and the pressure they experience is likely to have a negative impact on their quality of life. The same interpretation shall be given in the case of cohabitation, where married patients may experience difficulties in meeting family responsibilities and the demands of their treatment.

Emotional intelligence is the ability that can mitigate depression burden and lead to improving well-being [29]. Studies regarding emotional intelligence in peritoneal patients are very limited and were conducted involving patients on hemodialysis. The present study showed moderate levels of emotional intelligence. Training dialysis patients to acquire appropriate emotional intelligence skills helps them to reduce their anxiety levels [30]. Also, indirect benefits accrue to patients when nephrology nurses apply emotional intelligence skills [31]. Indeed, nurses, when properly trained, can implement educational programs for their patients, which provide them with the ability to improve patients' levels of emotional intelligence, both as a whole and in its domains. In turn, emotional intelligence contributes to improving patients' quality of life [32]. Follow-up results showed the advantages of these programs: their implementation going through nurses, are short in duration (30–45 min each session) with benefits seem to last over time. [32]. Further results demonstrated a positive correlation between emotional intelligence and quality of life, making it imperative to improve these patients' level of emotional intelligence. Not only do ESRD patients gain advantages from emotional intelligence, but also groups of patients with different chronic diseases or conditions [33]–[35].

The present study has a series of limitations. First, it refers to the fact that it is an observational cross-sectional study. Therefore, the association between body image, emotional intelligence and quality of life does not make a causal relation. Second, it was carried out in a relatively small number of participants, so the results cannot be generalized. Third, there are other demographic and clinical characteristics, such as the number of children, income, presence of comorbidities and creatinine clearance, that according to the literature affect the quality of life of patients on peritoneal dialysis and that were not included in this study. Therefore, the findings of this study should be interpreted with caution and cannot be generalized.

## 5. Conclusions

ESRD patients on peritoneal dialysis suffer significant limitations related to disease and treatment modality. These affect both their body image and their quality of life, according to the findings of this study. Although patients on peritoneal dialysis have better quality of life compared to those on hemodialysis, they too experience some deterioration in their life. Improvement in emotional intelligence becomes the factor that plays an important mediating role in improving both body image and quality of life in patients on peritoneal dialysis.

## Use of AI tools declaration

The authors declare they have not used Artificial Intelligence (AI) tools in the creation of this article.

## References

[1] Lv JC, Zhang LX (2019). Prevalence and disease burden of chronic kidney disease. Adv Exp Med Biol.

[2] USRDS (2022). Annual data report.

[3] Clarke LH, Griffin M (2008). Failing bodies: Body image and multiple chronic conditions in later life. Qual Health Res.

[4] Álvarez-Villarreal M, Velarde-García JF, Chocarro-Gonzalez L (2019). Body changes and decreased sexual drive after dialysis: A qualitative study on the experiences of women at an ambulatory dialysis unit in Spain. Int J Environ Res Public Health.

[5] Partridge KA, Robertson N (2011). Body-image disturbance in adult dialysis patients. Disabil Rehabil.

[6] Öyekçin DG, Gülpek D, Sahin EM (2012). Depression, anxiety, body image, sexual functioning, and dyadic adjustment associated with dialysis type in chronic renal failure. The International Journal of Psychiatry in Medicine.

[7] Megari K (2013). Quality of life in chronic disease patients. Health Psychol Res.

[8] Jung HY, Jeon Y, Park Y (2019). Better quality of life of peritoneal dialysis compared to hemodialysis over a two-year period after dialysis initiation. Sci Rep.

[9] Chen JY, Wan EYF, Choi EPH (2017). The health-related quality of life of Chinese Patients on hemodialysis and peritoneal dialysis. Patient.

[10] Salovey P, Mayer JD (1990). Emotional intelligence. Imagin, Cognition and Personality.

[11] Barberis N, Costa S, Gitto L (2016). Role of emotional intelligence as a mediating factor between uncertainty and anxiety hospital in chronic renal patients. Illn, Crisis Loss.

[12] Sadeghpour F, Heidarzadeh M, Naseri P (2021). Emotional intelligence as a predictor of posttraumatic growth in patients undergoing hemodialysis. Illn, Crisis Loss.

[13] Cash TF (2002). The situational inventory of body-image dysphoria: Psychometric evidence and development of a short form. Int J Eat Disord.

[14] Argyrides M, Kkeli N (2015). Validation of the factor structure of the Greek adaptation of the Situational Inventory of Body-Image Dysphoria-Short Form (SIBID-S). Eat Weight Disord.

[15] Stamatopoulou M, Galanis P, Prezerakos P (2016). Psychometric properties of the Greek translation of the Trait Emotional Intelligence Questionnaire-Short Form (TEIQue-SF). Pers Individ Dif.

[16] Petrides KV, Furnham A (2006). The role of trait emotional intelligence in a gender-specific model of organizational variables. J Appl Soc Psychol.

[17] Petrides KV, Assessing Emotional Intelligence (2009). Psychometric properties of the Trait Emotional Intelligence Questionnaire (TEIQue).

[18] Malindretos P, Sarafidis P, Spaia S (2010). Adaptation and validation of the kidney disease quality of life-short form questionnaire in the Greek language. Am J Nephrol.

[19] Salzer WL (2018). Peritoneal dialysis-related peritonitis: Challenges and solutions. Int J Nephrol Renovasc Dis.

[20] Morris A, Liles C, Roskell C (2015). Exploring the challenges of living with peritoneal dialysis. Journal of Renal Nursing.

[21] Tylka TL, Wood-Barcalow NL (2015). What is and what is not positive body image? Conceptual foundations and construct definition. Body Image.

[22] Partridge KA, Robertson N (2011). Body-image disturbance in adult dialysis patients. Disabil Rehabil.

[23] Güçer BK, Kantarcı G (2020). Body Image Perception of Chronic Kidney Disease Patients and Its Impact on Their Personal Relationships. Turk J Nephrol.

[24] Sharif Nia H, Kohestani D, Froelicher ES (2022). The relationship between self-care behavior and concerns about body image in patients undergoing hemodialysis in Iran. Front Public Health.

[25] Jung HY, Jeon Y, Park Y (2019). Better quality of life of peritoneal dialysis compared to hemodialysis over a two-year period after dialysis initiation. Sci Rep.

[26] Grace BS, Clayton PA, Gray NA (2014). Socioeconomic differences in the uptake of home dialysis. Clin J Am Soc Nephrol.

[27] Moisoglou I, Margariti E, Kollia K (2017). The role of demographic characteristics and comorbidities in hemodialysis patients' health-related quality of life. Hippokratia.

[28] Khatib ST, Hemadneh MK, Hasan SA (2018). Quality of life in hemodialysis diabetic patients: A multicenter cross-sectional study from Palestine. BMC Nephrol.

[29] Fernández-Berrocal P, Extremera N (2016). Ability emotional intelligence, depression, and well-being. Emot Rev.

[30] Yarahmadi F, Ghasemi FS, Forooghi S (2023). The effects of emotional intelligence training on anxiety in hemodialysis patients. J Nurs Midwifery Sci.

[31] Supramanian K, Shahruddin R, Sekar M (2021). Emotional intelligence using ability model in context of nursing and its impact on end-stage renal disease patients: A narrative review. Int J Curr Res Rev.

[32] Shahnavazi M, Parsa-Yekta Z, Yekaninejad MS (2018). The effect of the emotional intelligence education programme on quality of life in haemodialysis patients. Appl Nurs Res.

[33] Andrei F, Nuccitelli C, Mancini G (2018). Emotional intelligence, emotion regulation and affectivity in adults seeking treatment for obesity. Psychiatry Res.

[34] Costa J, Marôco J, Pinto-Gouveia J (2020). Depression and physical disability in chronic pain: The mediation role of emotional intelligence and acceptance. Aust J Psychol.

[35] Moradi F, Tourani S, Ziapour A (2020). Emotional intelligence and quality of life in elderly diabetic patients. Community Health Equity Res Policy.

